# *In vivo* functional properties of juxtaglomerular neurons in the mouse olfactory bulb

**DOI:** 10.3389/fncir.2013.00023

**Published:** 2013-02-21

**Authors:** R. Homma, Y. Kovalchuk, A. Konnerth, L. B. Cohen, O. Garaschuk

**Affiliations:** ^1^Department of Physiology, Yale UniversityNew Haven, CT, USA; ^2^NeuroImaging Cluster, Marine Biological LaboratoryWoods Hole, MA, USA; ^3^Institute of Physiology II, University of TuebingenTuebingen, Germany; ^4^Institute of Neuroscience, Technical University MunichMunich, Germany

**Keywords:** *in vivo* calcium imaging, olfaction, odor-evoked responses, mammalian olfactory bulb

## Abstract

Juxtaglomerular neurons represent one of the largest cellular populations in the mammalian olfactory bulb yet their role for signal processing remains unclear. We used two-photon imaging and electrophysiological recordings to clarify the *in vivo* properties of these cells and their functional organization in the juxtaglomerular space. Juxtaglomerular neurons coded for many perceptual characteristics of the olfactory stimulus such as (1) identity of the odorant, (2) odorant concentration, (3) odorant onset, and (4) offset. The odor-responsive neurons clustered within a narrow area surrounding the glomerulus with the same odorant specificity, with ~80% of responding cells located ≤20 μm from the glomerular border. This stereotypic spatial pattern of activated cells persisted at different odorant concentrations and was found for neurons both activated and inhibited by the odorant. Our data identify a principal glomerulus with a narrow shell of juxtaglomerular neurons as a basic odor coding unit in the glomerular layer and underline the important role of intraglomerular circuitry.

## Introduction

The mammalian olfactory epithelium consists of a single layer of non-interacting olfactory receptor neurons (ORNs). Each ORN typically expresses only one type of olfactory receptor protein (Chess et al., 1994; Serizawa et al., 2000), which defines its odorant selectivity. Axons of thousands of ORNs expressing the same olfactory receptor protein converge onto a few (usually two) discrete glomeruli in one olfactory bulb (Vassar et al., 1994; Mombaerts et al., 1996). It is in the bulb that the first stage of olfactory processing occurs. In the glomeruli the ORN axons synapse on the principal mitral/tufted neurons of the bulb and on local interneurons. There are three major morphologically distinct classes of local interneurons in the glomerular layer-periglomerular cells, short-axon cells, and external tufted cells-they are collectively referred to as juxtaglomerular neurons (Pinching and Powell, 1971; Kosaka and Kosaka, 2007; Parrish-Aungst et al., 2007). The juxtaglomerular neurons have rich synaptic connections with each other. In addition, they target both input [ORN axon terminals (Aroniadou-Anderjaska et al., 2000; McGann et al., 2005; Murphy et al., 2005)] and output (mitral/tufted) neurons of the bulb.

In mice about half of the juxtaglomerular neurons are GABAergic [as shown by combining GAD65-GFP mice and antibodies directed against glutamate decarboxylase GAD67; (Parrish-Aungst et al., 2007)]. These GABAergic cells presynaptically inhibit glutamate release from ORN terminals, and also mediate postsynaptic inhibition of external tufted and mitral/tufted cells (Aroniadou-Anderjaska et al., 2000; Murphy et al., 2005). *In vitro* evidence suggests that periglomerular cells have a lower activation threshold compared to mitral cells (Gire and Schoppa, 2009). By means of feed forward inhibition they can prevent ORN-induced firing of mitral cells at low stimulus strength.

Excitatory juxtaglomerular neurons (i.e., external tufted cells) are implicated in feedforward excitation of mitral cells (De Saint Jan et al., 2009). The activation threshold of these cells is also lower than that of mitral cells (Gire and Schoppa, 2009) and therefore they are in a position to integrate the inputs from ORNs and inhibitory periglomerular neurons before signaling to output neurons. Interestingly, firing of a single external tufted cell is sufficient to activate mitral cells belonging to the same glomerulus (De Saint Jan et al., 2009). Taken together these and other (Dhawale et al., 2010; Fukunaga et al., 2012; Gire et al., 2012) data suggest that mitral cells are mainly excited through an indirect signaling pathway critically involving both excitatory and inhibitory juxtaglomerular neurons.

Despite the wealth of *in vitro* data, relatively little is known about odorant-evoked responses of mammalian juxtaglomerular neurons. The first *in vivo* recordings were obtained with intracellular microelectrodes (Wellis and Scott, 1990; Scott, 1991) but these included only four juxtaglomerular neurons. All four cells responded with bursts of action potentials to the onset of odorant presentation. Adaptation/desensitization of the response to a repeated odorant presentation was observed in three of the four recorded cells. Three of the cells were spontaneously active and one was silent. More recent studies have shown that juxtaglomerular neurons show odorant-induced increases in the intracellular calcium concentration [[Ca^2+^]_*i*_; (Petzold et al., 2008)] and respond to a large number of different odorants thus having broad tuning curves (Tan et al., 2010).

For characterization of *in vivo* odorant response properties of juxtaglomerular neurons, we combined the multi-cell bolus-loading technique (Stosiek et al., 2003; Garaschuk et al., 2006b), two-photon calcium imaging, and simultaneous loose-seal cell-attached recordings of spike activity. We monitored spontaneous and odor-evoked activity of these cells, assessed the odorant response properties of dozens-to-hundreds of individual neurons in each preparation and determined the spatial locations of responding cells with respect to the odorant-responsive glomeruli.

## Materials and methods

### Animals and ethical approval

All animal care and experimental procedures were performed in accordance with institutional animal welfare guidelines and were approved by the Institutional Animal Care and Use Committee of the Marine Biological Laboratory, USA and by the state government of Baden-Württemberg, Germany.

C57BL/6 mice of both sexes (*n* = 39), 25–35 days old, were anesthetized by intraperitoneal injection of a mixture of Ketamine/Xylazine (80/8 or 80/4 μg/g of body weight for the initial injection). Anesthetic depth was monitored by toe pinch and additional Ketamine/Xylazine (40/2 μg/g of body weight) was injected when necessary. Data acquisition started at least 10 min after the last injection of the anesthetics. The animal breathed freely throughout the experiment. The respiration rate was monitored using a pressure sensor attached to the body of the animal and we attempted to keep it between 160 and 200 cycles per minute. Pure oxygen gas was supplied to maintain the animal's physiological state when the respiration rate fell below 100 per minute. The rectal temperature was kept between 36.5°C and 38°C.

The skin covering the dorsal part of the olfactory bulb was trimmed following a subcutaneous injection of local anesthetic (2% lidocaine or 0.5% bupivacaine) and a recording chamber with a hole in the center (Garaschuk et al., 2006b) was attached with cyanoacrylic glue to the skull so that the hole was over the recording site in the olfactory bulb. For recordings the chamber was fixed on a microscope stage. The skull above the dorsal bulb was thinned using a dental drill; the recording chamber was perfused with standard extracellular solution (in mM: 125 NaCl, 4.5 KCl, 26 NaHCO_3_, 1.25 NaH2PO_4_, 2 CaCl_2_, 1 MgCl_2_, and 20 glucose, pH 7.4 when bubbled with 95%O_2_ and 5%CO_2_) warmed to 38°C. A craniotomy (typical size about 1 × 0.5 mm) was made using a 30 gauge syringe needle. The dura was left intact.

### Multi-cell bolus-loading

The multi-cell bolus-loading technique was described previously (Stosiek et al., 2003; Garaschuk et al., 2006b). In brief, 10 mM Oregon Green 488 BAPTA-1 AM (Invitrogen, Carlsbad, CA) or Fura PE-3 AM (TEFLAB, Austin, TX) was prepared by dissolving the dye powder in 20%w/v Pluronic F-127 (Invitrogen) in DMSO. Then the mixed solution was diluted 10–50 times with Ca^2+^ and Mg^2+^ free pipette solution (in mM: 150 NaCl, 2.5 KCl, and 10 HEPES, pH 7.4). The dye solution was injected into the brain tissue through a patch pipette (tip diameter ~1 μm) using a Picospritzer II (8–10 psi for 1–2 min; General Valve, Fairfield, NJ). We preferred to use Fura PE-3 AM when imaging mice expressing monomeric red fluorescent protein (mRFP1) and/or AmCyan1 under the astrocyte-specific glial fibrillary acidic protein promoter (Figure A2). Because Fura PE-3 AM does not fluoresce when illuminated at 930 nm (Xu, 2000), the use of this dye provided better color separation in those mice. We injected dyes at one to four sites in order to stain most of the area under the cranial window. The chamber opening was then covered with 2% agarose to suppress movement artifacts. We could record calcium signals for up to 8 h. In several initial experiments, the dye solution also contained the cortical astrocyte marker sulforhodamine 101 (Sigma-Aldrich, St. Louis, MO, 0.023%w/v final concentration). Using a protocol which reliably stains cortical astrocytes (Nimmerjahn et al., 2004; Garaschuk et al., 2006b), we were unable to find clearly labeled cells in the olfactory bulb.

### Nose-loading with dextran dye

The ORNs were stained with the fluorescent dye Alexa Fluor 594-dextran (Mw 10,000, Invitrogen) following the methods described in Wachowiak and Cohen (2001). The animal was anesthetized by intraperitoneal injection of a mixture of Ketamine/Xylazine (80/8 μg/g of body weight). The dye solution was composed of 4%w/v dye and 0.06%v/v Triton X-100 dissolved in distilled water. 10 μl of dye solution was slowly injected in two portions a few minutes apart through a microloader pipette tip (Eppendorf, Westbury, NY) inserted into the nostril. The animal was used for the two-photon recording 4 or 5 days after the nose injection.

### Two-photon imaging

**Figure 1:**
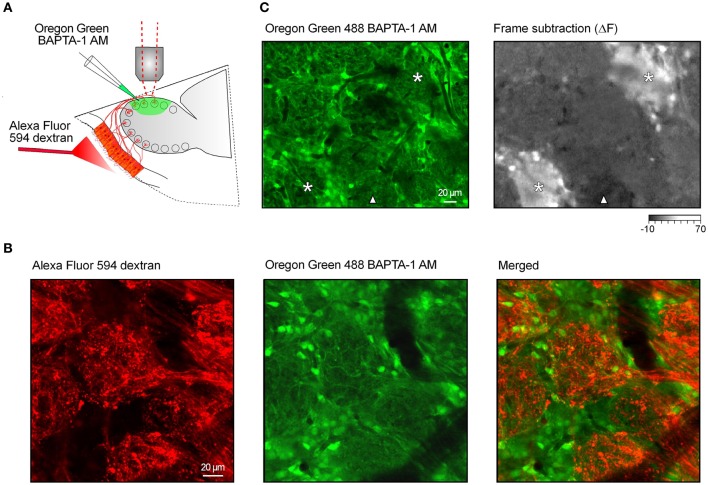
**Functional calcium imaging of the juxtaglomerular neuronal network. (A)** Schematic drawing of the experimental arrangement. Cells and processes in the glomerular layer were stained with a calcium indicator Oregon Green 488 BAPTA-1 AM [OGB-1, (green)]. In a few preparations the fluorescent marker Alexa Fluor 594 dextran (red) was injected into the nasal cavity to stain the axons of the olfactory receptor neurons (see **B**). **(B)** *In vivo* images of the stained tissue. The fluorescence of Alexa Fluor 594 dextran (left panel) and OGB-1 (middle panel) was split using a 570 nm dichroic mirror. Right panel, an overlay of the two images. **(C)** Screening for locations with robust odorant responses using a low magnification (20x) objective lens. Left panel, an image of the glomerular layer labeled with OGB-1. Right panel, a frame subtraction image obtained by subtracting the average of nine frames recorded before the odorant presentation from a similar image recorded during the odorant presentation (2-hexanone, 1.5% of saturated vapor). Asterisks mark two odor-activated glomeruli; a triangle marks a glomerulus showing an odorant-induced decrease in OGB-1 fluorescence. The intensity scale is shown in arbitrary units (AU).

Two-photon imaging was performed with a custom-built two-photon laser-scanning microscope based on a mode-locked laser operating at 690–1040 nm wavelength (MaiTai, Spectra Physics, Mountain View, CA) and a laser-scanning system (Olympus Fluoview 300, Olympus, Tokyo, Japan) coupled to an upright microscope (BX51, Olympus, Tokyo, Japan) and equipped with one of the following water-immersion objectives: a 16x N.A. 0.80 (Nikon, Melville, NY), a 20x N.A. 1.0 (Zeiss, Thornwood, NY), a 40x N.A. 0.80 (Nikon), or a 60x N.A. 1.0 (Nikon). 16x and 20x objectives were used for large-scale imaging (e.g., Figures 1B,C) whereas 40x and 60x objectives were used for higher-resolution imaging of individual neurons (e.g., Figure 3A). For each experiment we selected an optimal objective providing an image of a required field of view along with maximum number of pixels per imaging structure and maximal possible acquisition speed. All of the results come from single trial recordings; signal averaging was not used. Calcium indicator dyes were excited at 800 nm. The sampling rate was 5–10 frames/s. To achieve higher temporal resolution, we also used line scanning at 200 Hz.

### Loose-seal cell-attached recordings *in vivo*

Loose-seal recordings were performed using an EPC-10 patch clamp amplifier (HEKA, Lamprecht, Germany) as described in Rochefort et al. (2009). The recording pipettes had a resistance of 3–6 MOhm when filled with a pipette solution containing (in mM): 150 NaCl, 2.5 KCl, 10 HEPES, 2 CaCl_2_, 1 MgCl_2_, and 20 glucose, 0.05 Alexa Fluor 594, pH 7.4. All data were filtered at 3 kHz and digitized at 20 kHz. Spike frequencies of neurons were calculated offline as reciprocals of inter-spike intervals.

### Odorant delivery

**Figure 2:**
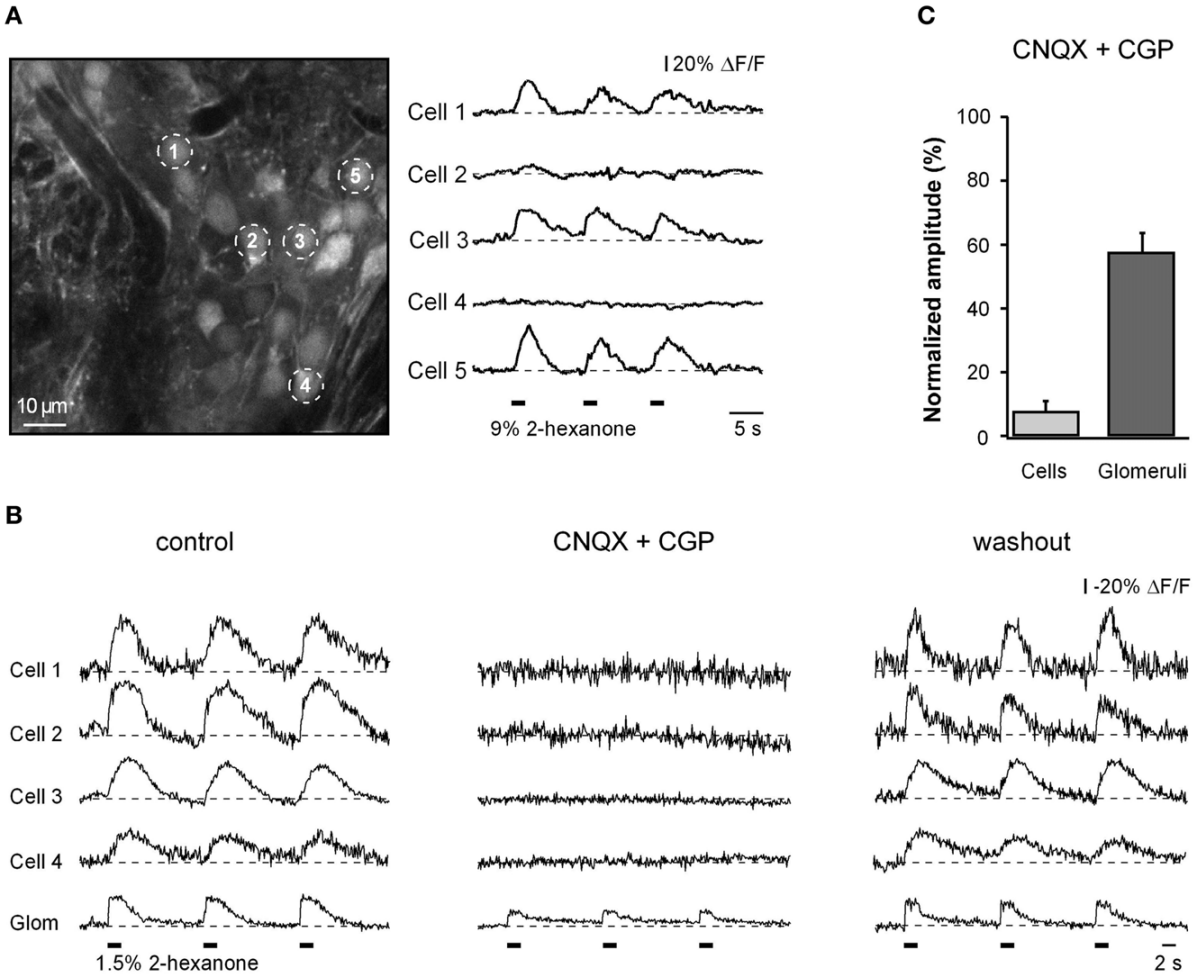
**Cellular and glomerular odor-evoked calcium signals. (A)** ΔF/F traces (right) recorded from juxtaglomerular neurons marked with respective numbers in the left panel. In this and subsequent figures the timing of odorant presentations is indicated as thick black bars below the traces. Note that only a fraction of cells (1, 3, and 5) showed odor-evoked Ca^2+^ responses. **(B)** ΔF/F traces recorded from four different cells and the corresponding glomerulus in another experiment before (left), during (middle), and after (right) application of 50 μM CNQX (AMPA receptor blocker) and 100 μM CGP (NMDA receptor blocker) to the surface of the olfactory bulb. **(C)** Summary of the data obtained in six different experiments (*n* = 26 cells, six glomeruli). For each cell/glomerulus the amplitude (mean of three trials) measured in the presence of the drugs was normalized to the mean amplitude in control. The odorant was 2-hexanone.

In an individual trial (e.g., Figure 2A), three pulses of odorant were applied through a custom-made flow-dilution olfactometer (Vucinic et al., 2006). The typical duration of odorant pulses was 2 sec and the inter-stimulus intervals were 8 sec. These odorant pulses are briefer than the ones previously shown to cause sensory-evoked Ca^2+^ transients in astrocytes [stimulus duration >10 s (Petzold et al., 2008)]. The odorant delivery was not timed relative to respiration. The odorants were monomolecular compounds that were known to evoke activity in dorsal glomeruli (Wachowiak and Cohen, 2001; Vucinic et al., 2006). In many experiments we tested five odorants; the most commonly used five were 2-hexanone, isoamyl acetate, hexanal, benzaldehyde, and propanal. All the odorants were purchased from Sigma-Aldrich and of highest commercially available purity. Apart from recording dose-response relationships we routinely used two different odorant concentrations: 9% and 1.5% of saturated vapor. These concentrations are in the upper half of the concentration range where rodents reliably recognize odorants (Homma et al., 2009).

### Drug application

Pharmacological substances were added to the extracellular saline perfusing the chamber attached to the mouse's skull (Garaschuk et al., 2006b). CNQX (6-Cyano-7-nitroquinoxaline-2,3-dione) and CGP-37849 [(E)-(±)-2-Amino-4-methyl-5-phosphono-3-pentenoic acid] were purchased from Ascent Scientific (Cambridge, UK).

### Data analysis

Data inspection during the experiment was carried out using Fluoview (Olympus, Tokyo, Japan) and NeuroPlex (RedShirtImaging LLC, Decatur, GA) software. Detailed data analysis was performed off-line using a combination of Fluoview, NeuroPlex, ImageJ (http://rsb.info.nih.gov/ij/) with WCIF plug-in (Wright Cell Imaging Facility, Toronto, Canada), MetaMorph (Molecular Devices, West Chester PA), and Excel (Microsoft, Redmond, WA), as well as custom made routines written for Labview (National Instruments, Austin, TX), Igor Pro (WaveMetrics, Portland, OR), or IDL (ITT, Boulder, CO).

Two complementary methods of initial data analysis were used. In the first method, responsive cells were found using a frame subtraction procedure. Ten to twenty consecutive frames (1–2 sec in actual duration) corresponding to the peak of odor-evoked calcium responses were averaged. A similar number of frames taken from the period just before the odorant presentation were averaged and then subtracted from the average during the response. The resulting subtracted image highlights the pixels showing a change in fluorescence (either negative or positive) in response to an odorant (e.g., Figure 1C). To search for OFF responses, the frame subtraction was performed between the periods just after and just before the stimulus offset. The group of responding pixels with the size of a cell or glomerulus was identified as a responsive cell or glomerulus and time courses of fluorescence intensity from those regions were used for further analysis. Frame subtractions were performed with NeuroPlex software.

In the second method, the locations of all the cell bodies were extracted manually from an image made by averaging all the frames in a trial. The fluorescence signals from the regions of interest covering each cell were then examined for odorant responses. In this manual extraction all identifiable cells and glomeruli were delineated using ImageJ. The pixel values from the delineated area of each cell or glomerulus were retrieved from each frame. A custom made Igor Pro routine was used for the automatic detection of odor-evoked Ca^2+^ transients in individual neurons and in the glomerular neuropil. First, background fluorescence, measured in blood vessel lumen, was subtracted from all signals. Second, all signals were expressed as relative fluorescence changes (ΔF/F). For automatic detection of responding cells all ΔF/F traces were smoothed with a binomial filter (time window 0.3 s). Each smoothed trace was subtracted from the original ΔF/F trace, resulting in the “baseline noise” trace. Calcium transients were automatically detected with a template-matching algorithm, taking into account their sharp rise. They were accepted as signals if their amplitude was three times larger than the standard deviation of the corresponding baseline noise values. After the automatic analysis, all traces were carefully inspected again to correct for false positive or negative signals. When analyzing neuronal activity, a threshold ΔF/F value of 15% was set in order to avoid contamination by responses of glial cells (Figure A2). This threshold also excluded a relatively small number of weakly-responding neurons causing an underestimation of the number of responding ON and OFF juxtaglomerular neurons. This threshold was not applied to INHIBITED signals. However, INHIBITED signals may also be under-reported because of the noise in our measurements (typically a ΔF/F of 5–10%). We used either OGB-1 or Fura PE-3 for Ca^2+^ imaging. An increase in [Ca^2+^]_*i*_ results in signals in opposite directions for the two dyes. The traces are presented such that an increase in calcium concentration is shown as an upward deflection (e.g., as ΔF/F for experiments performed using OGB-1 and as −ΔF/F for experiments performed using Fura PE-3).

When a data set was analyzed with both methods the results were largely consistent. The frame subtraction identified the responsive cells rapidly and occasionally identified cells that were difficult to discern from the image. On the other hand, it failed to identify cells with small signals and it does not provide information about the number of non-responsive cells.

The 3-D volume reconstructions of cells and glomeruli were done using Fiji (http://pacific.mpi-cbg.de/wiki/index.php/Fiji). The same program was used to measure the distance of the cell to the three-dimensionally nearest odor-activated glomerulus. Histograms summarizing the position of odor-responsive neurons (e.g., Figures 9E,F) were constructed separately for each experiment and then were averaged together.

#### Temporal and spatial filtering

In Figure A2B (inset) the time course data was temporally smoothed with a binomial (1-2-1) low-pass filter. Temporal smoothing was not used for any other traces shown. For the frame subtraction images (Figure 1C) we used two passes of a spatial low-pass filter that replaced each pixel with the mean of a 3 × 3 pixel region surrounding the pixel.

All measured values are given as mean ± SEM.

## Results

### *In vivo* identification of active cells and glomeruli

We stained the cells and processes in a 500 μm by 1 mm area of the dorsal olfactory bulb of mice with a calcium indicator dye. We have used either Oregon Green 488 BAPTA-1 AM (OGB-1 AM; Figure 1, green) or Fura PE-3 AM. The stained tissue included the cell bodies of the juxtaglomerular neurons and astrocytes as well as the processes of juxtaglomerular neurons, mitral/tufted cells, glial cells, and ORNs. In addition, in a few preparations a bright fluorescent dye, Alexa Fluor 594 dextran, was injected into the nose to stain the axons and terminals of ORNs [Figures 1A,B, red; (Wachowiak and Cohen, 2001)]. In such double-labeled preparations, staining with Ca^2+^-sensitive dyes revealed regions with densely packed cell bodies and darker, usually cell body free, oval-shaped regions (Figure 1B, middle panel, Figure 1C left panel). The terminals of ORNs labeled with Alexa Fluor 594 (Figure 1B, left panel) were found in the cell body free oval-shaped regions in the merged image (Figure 1B, right panel) thereby identifying these as glomeruli. We examined 117 double-labeled glomeruli in four mice and concluded that the Ca^2+^-sensitive dye staining alone allows unequivocal identification of glomeruli as well as juxtaglomerular regions.

We began by examining the stained area (5–15 glomeruli) at low magnification (Figures 1B,C). We did not observe any spontaneous Ca^2+^ transients when recording from the neuropil of individual glomeruli. The only “spontaneous” transients we detected were strictly correlated with infrequently occurring deep breaths of the animal. These were not studied further. To identify locations with robust odorant responses we averaged the frames recorded during the 2 sec odorant stimulation and subtracted an equal number of frames just prior to the odor presentation. In the example illustrated in Figure 1C the frame subtraction image (right panel) reveals two separate glomeruli and surrounding cells with relatively large increases in [Ca^2+^]_*i*_ (asterisks) as well as a location (marked with a triangle) where the odorant caused a small decrease in the OGB-1 signal. Locations with robust odorant responses were used for subsequent measurements at higher magnification (thus restricting the imaged field to 2–6 glomeruli in one plane of focus).

### Odorant-evoked calcium signals in juxtaglomerular neurons

At locations with robust odorant responses, odorant presentations caused Ca^2+^ transients in many individual juxtaglomerular neurons (Figure 2A). Most often, the cells showed a transient increase in [Ca^2+^]_*i*_ in response to odorant presentation. Such responses were termed odor-activated responses. The majority of odor-activated cells responded at the onset of odorant presentation (ON responses; Figure 2A). As expected, odor-activated responses in individual juxtaglomerular neurons were strongly and reversibly blocked by a mixture of glutamate receptor blockers (CNQX and CGP) whereas the Ca^2+^ signals in the nearby glomeruli, which consists of both pre- and postsynaptic components, decreased only partially (Figures 2B,C). For glomeruli with ON responses, 58% of the glomerular signal remained after drug treatment and was presumed to arise from the nerve terminals of the receptor neurons and 42% was contributed by post-synaptic processes (Figure 2C).

**Figure A1:**
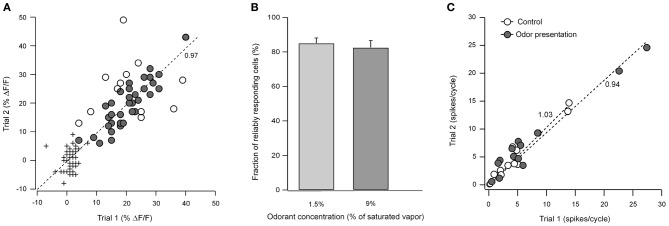
**Consistency of odorant-evoked responses in repeated trials. (A)** Amplitudes of odor-evoked neuronal Ca^2+^ transients recorded in two different trials (circles) plotted against each other. The crosses depict the noise level in the corresponding traces. Dark circles mark cells whose response amplitudes changed by an amount smaller than two standard deviations of the noise level (reliably responding cells), the open circles mark cells with a larger variation in response amplitude. The odorant was 2-hexanone at 9% of saturated vapor. The dashed line represents the linear least-squares fit of the data. **(B)** Fraction of reliably responding cells at two different odorant concentrations (*n* = 88 cells, 5 experiments and *n* = 201 cells, 8 experiments for 1.5, and 9% of saturated vapor, respectively). Odorants were: 2-hexanone (*n* = 5), hexanal (*n* = 1), benzaldehyde (*n* = 2), heptanal (*n* = 4), isoamyl acetate (*n* = 1). **(C)** Spike rates from loose-patch recordings recorded in two different trials (measured as a number of action potentials per respiratory cycle) before (open circles) and during (filled circles) odorant presentations plotted against each other (*n* = 4, 5, and 4 trial pairs for 0.3, 1.5, and 9% of saturated vapor). The odorant was 2-hexanone (*n* = 8 cells). Both lines represent the linear least squares fits to the respective set of data. Note that the slope of each fit line in (**A** and **C**) (the number near the line) is close to 1 suggesting that there are little or no differences in the mean response between the two trials.

To test the reproducibility of the odorant responses in individual juxtaglomerular neurons we carried out two trials with identical odorant stimulations in 13 instances. Each trial contained three 2-second-long odorant applications applied at an inter-stimulus interval of 8 sec (e.g., Figure 2A right panel). The open and filled circles in Figure A1A illustrate the results of one such measurement pair. Here we have plotted the signal amplitude from the first odorant application in trial 1 vs. the corresponding amplitude in trial 2 for 44 cells. We considered the cells as responding reliably if the trial-to-trial differences in the amplitude of their odor-evoked Ca^2+^ signals were within two standard deviations of the measurement noise. The noise level was calculated as the difference between the fluorescence intensity averaged over two time windows of 1 sec each taken from the pre-stimulus period (these values are shown as the + symbols near the origin in Figure A1A). Note that the slope of the line is close to one. The mean slope from 13 experiments was 0.97 ± 0.03 suggesting that there are little or no differences in the mean response between the two trials. In the illustrated experiment 73% of the cells had reliable odorant responses. The means from similar data in 12 additional experiments using odorants at 1.5% or 9% of saturated vapor are shown in Figure A1B. Thus, the majority of juxtaglomerular cells show trial-to-trial variability that is less than the noise in our measurements.

### Separation of neuronal and glial signals

**Figure A2:**
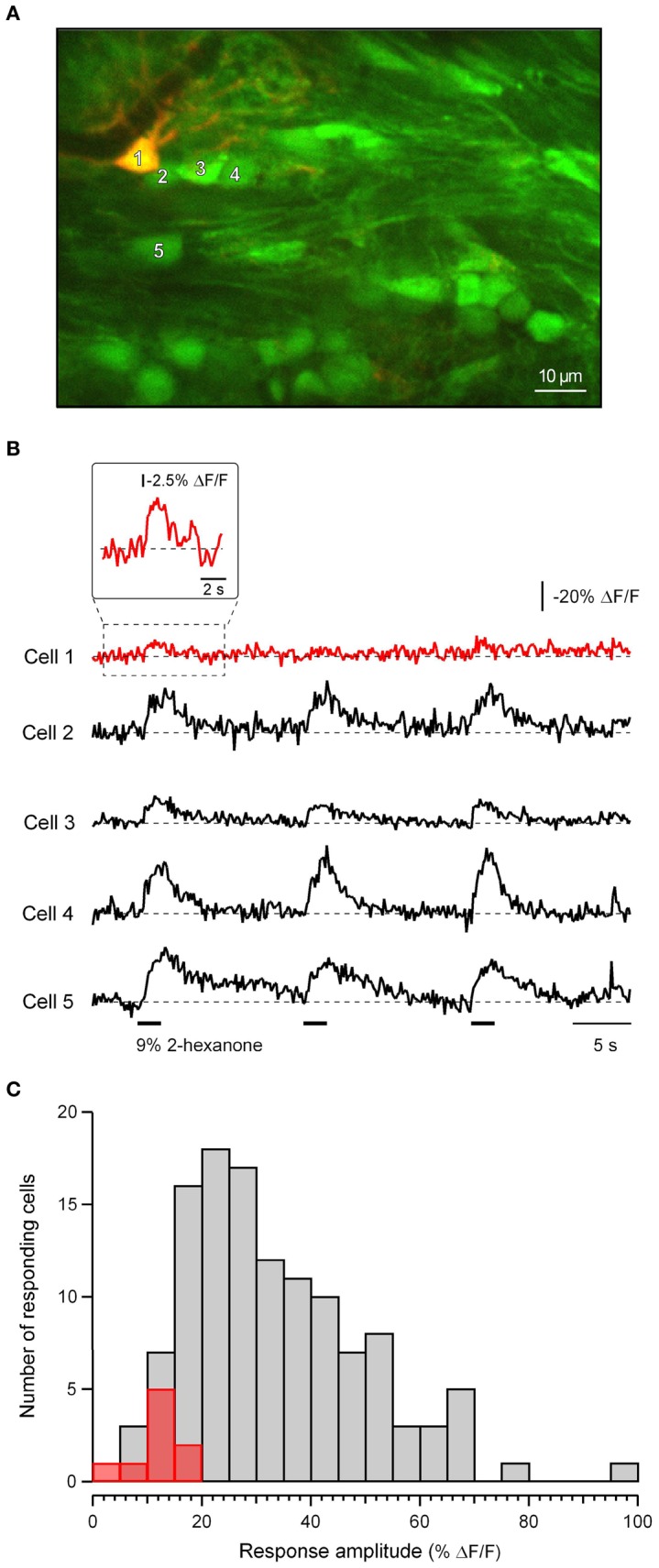
**Discrimination of neuronal and glial responses. (A)** A merged image of a mRFP1-positive glial cell (red, excited at 950 nm) in the area stained with Fura PE-3 AM (green, excited at 800 nm). **(B)** Recordings of [Ca^2+^]_*i*_ from the identified glial cell (cell 1) and the four mRFP-negative cells (cells 2–5) marked with numbers in **(A)**. The inset above the trace for cell 1 is an expansion of the boxed portion of the trace temporally smoothed with a binomial (1-2-1) low-pass filter. **(C)** Distribution of the amplitudes of odor-evoked responses of nine identified glial cells (red) and an additional 122 mRFP1/AmCyan1-negative cells from the same frames (gray). No odorant responses were detected in additional 22 glial cells.

In the olfactory bulb both neurons and astrocytes were shown to respond to 10-second-long presentations of odorants with an increase in [Ca^2+^]_*i*_ (Petzold et al., 2008). To estimate the possible contribution of astrocytes to Ca^2+^ signals measured in our study (using 2-second-long odorant presentations) we conducted a series of experiments in mice expressing monomeric red fluorescent protein (mRFP1) and/or AmCyan1 under the astrocyte-specific glial fibrillary acidic protein promoter (Hirrlinger et al., 2005). We chose this method because astrocytes in the olfactory bulb were not labeled with the astrocytic marker sulforhodamine 101 (Nimmerjahn et al., 2004). Twenty-two out of thirty-one astrocytes tested (*n* = 6 mice) failed to respond to the odorant application under our experimental conditions even though nearby non-labeled cells did respond. Seven of the nine remaining cells had ΔF/F signals smaller than 15%; only two had signals between 15% and 20% (Figures A2B,C). In contrast, 92% of 122 responding mRFP1/AmCyan1-negative cells in the analyzed frames (e.g., cells 2–5 in Figure A2A) had odorant responses larger than 15% (median = 33%; Figure A2C). Thus, in further analyses we have only included cells with odorant-evoked increases in [Ca^2+^]_*i*_ larger than 15%. The vast majority of these cells are likely to be neurons. Decreases in [Ca^2+^]_*i*_ were not seen in any of the labeled glial cells and have not been reported elsewhere and thus this 15% threshold was not used for cells showing odor-evoked decreases in [Ca^2+^]_*i*_.

### Electrical activity underlying ON responses in juxtaglomerular neurons

To obtain insight into electrical activity underlying Ca^2+^ signals in cells with ON responses, we carried out simultaneous two-photon and targeted loose-patch recordings from 20 ON neurons. Despite difficulties caused by dense cell packing, targeted patching under optical control (Margrie et al., 2003; Garaschuk et al., 2006a) enabled stable loose-seal recordings lasting 40–60 min.

**Figure 3:**
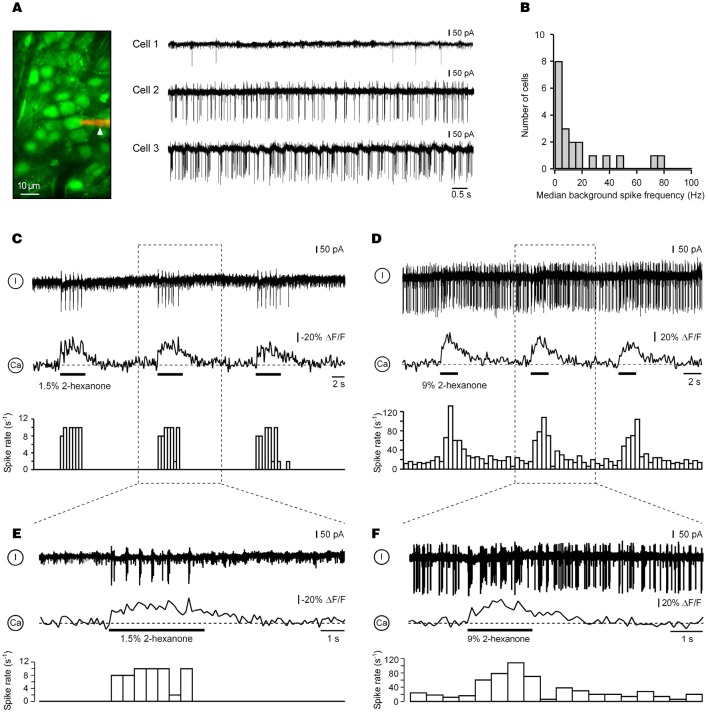
**Electrical activity underlying ON calcium responses in juxtaglomerular neurons. (A)** Right: background action potentials of juxtaglomerular neurons recorded in the loose patch mode before odorant presentation. The image in the left panel shows a patch pipette filled with 50 μM of Alexa Fluor 594 (arrowhead) attached to Cell 2. The three cells were recorded in different experiments. **(B)** Distribution of the median background spike frequencies of all recorded cells (*n* = 20). **(C,D)** Simultaneous Ca^2+^ imaging and loose-seal cell-attached recordings from two different odor-activated neurons. The top trace is the electrical recording, the middle trace the calcium signal, and the bottom trace the peristimulus time histogram obtained in the same trial. The experiment in **(C)** was performed using Fura PE-3 and the one in **(D)** using OGB-1. Boxed areas are expanded in **(E,F)**, respectively. The odorant was 2-hexanone.

**Figure A3:**
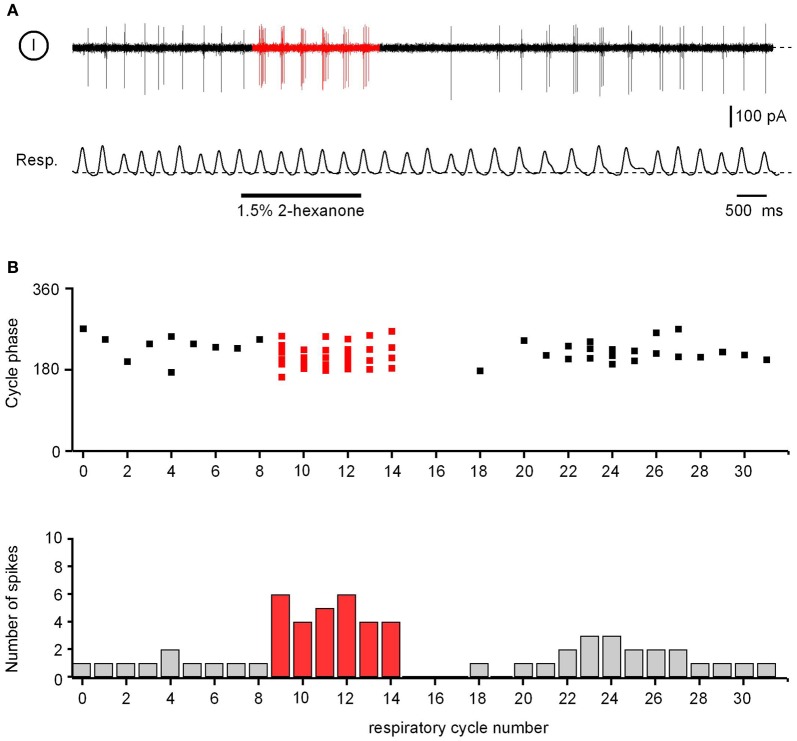
**Spontaneous and odor-evoked activity in ON juxtaglomerular neurons. (A)** Action potential firing of a juxtaglomerular neuron (top) recorded simultaneously with the respiration of the mouse (bottom). **(B)** Top: distribution of spikes (the same experiment as in **A**) relative to the phase of each respiratory cycle. Bottom: peristimulus time histogram illustrating the number of spikes fired during each respiratory cycle. (**A** and **B**) are not aligned.

Eight of the recorded cells were either absolutely quiet at rest (*n* = 2 cells) or had rather low median frequencies of background action potentials (0.3–4.9 Hz, *n* = 6; e.g., Cell 1 in Figure 3A). The remaining 12 ON neurons had median spiking frequencies ranging from 6 to 77 Hz (Figure 3B). In spiking neurons (*n* = 18), the peak instantaneous spike frequencies in the absence of the odorant also were substantially different from cell to cell, ranging from 40 to 420 Hz. In 92% of cells (*n* = 12/13) in which both background spike activity and mouse respiration were monitored in parallel (e.g., Figure A3A), the temporal pattern of the background spike activity was non-uniform across the respiratory cycle [chi-square test, 36 bins (bin size = 10°); *p* < 0.01]. Rather, it was locked to a specific phase of the respiratory cycle (Figure A3).

An application of an odorant caused an increase in the spike frequency of all ON cells tested (instantaneous peak spike frequencies: 210–460 Hz; *n* = 20) preserving its respiratory patterning (e.g., Figure A3A). This increase was directly associated with an increase in [Ca^2+^]_*i*_ (Figures 3C–F). Importantly, the amplitudes of odor-evoked Ca^2+^ transients were not altered by establishing the loose-seal recording conditions (the median ratio of amplitudes of odor-evoked Ca^2+^ transients recorded before and during electrical recordings was 0.98, *n* = 11, *p* = 0.4, Mann–Whitney Test). To access the reliability of spiking responses underlying such Ca^2+^ signals, we analyzed the number of action potentials per respiration cycle (spike rate) before and during presentation of the odorant in repeated trials. Consistent with the imaging data (Figures A1A,B), the spike rates of 10 different cells examined at 3 different odor concentrations (0.3%, 1.5%, and 9% of saturated vapor) were reproducible from trial to trial (Figure A1C).

### Juxtaglomerular neurons with OFF and inhibited responses

**Figure 4:**
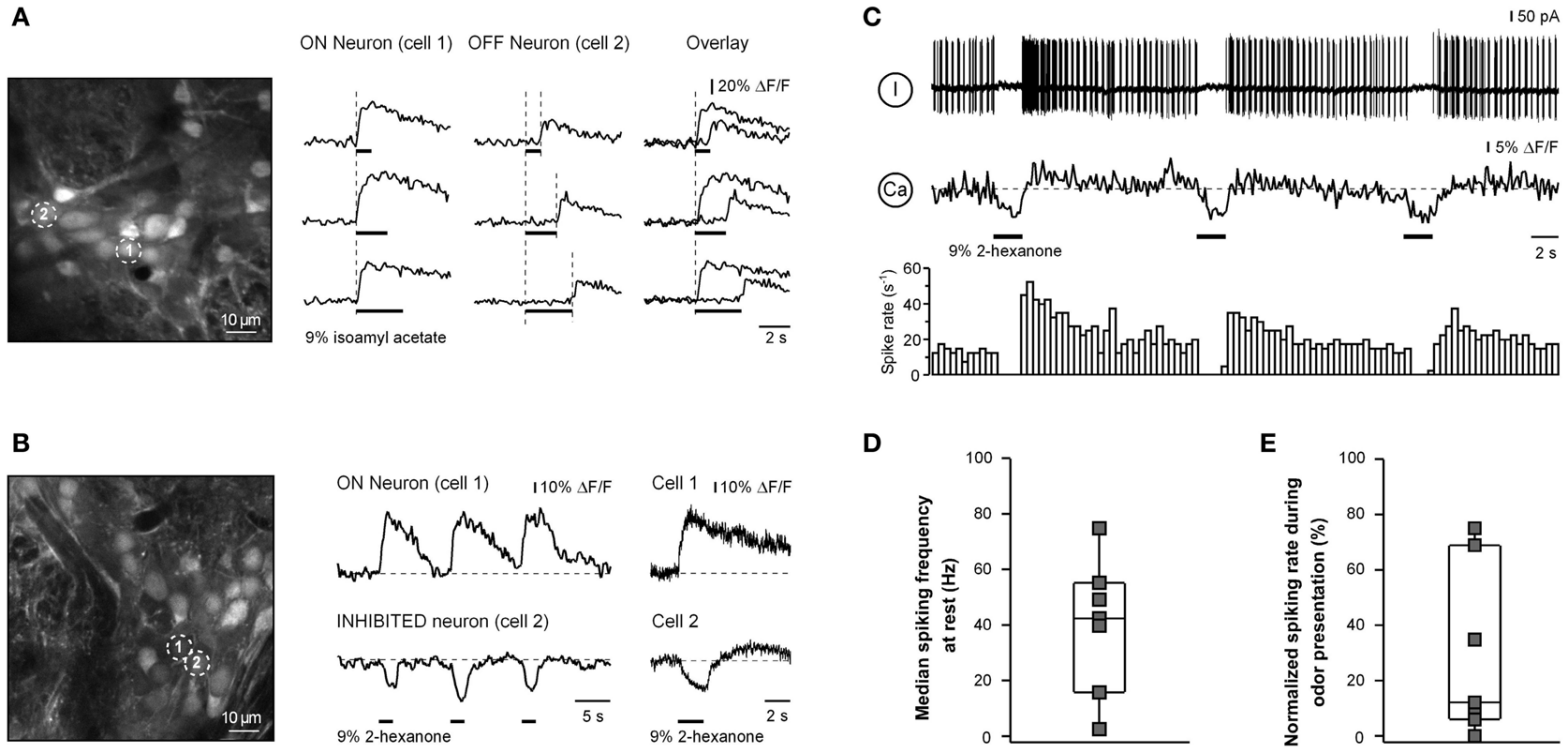
**OFF and INHIBITED responses in juxtaglomerular neurons. (A)** ΔF/F traces (right) recorded from two odor-responsive neurons marked with numbers in the image (left). In cell 1 [Ca^2+^]_*i*_ increases at the onset of the stimulus (ON response) while in cell 2 [Ca^2+^]_*i*_ increases at the offset of the odorant presentation (OFF response). Overlaid traces are shown in the right column. An increase in the duration of odorant presentation (as indicated by black bars of different length) caused a corresponding increase in the latency of the OFF response, showing that the cell responded to the offset of odorant rather than the onset of odorant with a delay. The odorant was isoamyl acetate. **(B)** ΔF/F traces (middle, right) recorded from neurons marked with numbers in the left panel (the same experiment as the one shown in Figure 2A). In cell 1 [Ca^2+^]_*i*_ increases at the onset of the stimulus (ON response) while in cell 2 [Ca^2+^]_*i*_ decreases in response to the odorant presentation (INHIBITED response). The right panel shows responses of the two neurons in a subsequent trial sampled at a higher temporal resolution using the line scan mode (200 Hz). Here and in **(C)** the odorant was 2-hexanone. **(C)** Simultaneous Ca^2+^ imaging and loose-seal cell-attached recording from another neuron with an INHIBITED response. The top trace is the loose-seal cell-attached recording, the middle trace is the calcium signal, and the bottom trace is the peristimulus time histogram. **(D)** Box plot illustrating median background spike frequencies of neurons with inhibited responses to odorants measured prior to odorant presentation (*n* = 7). **(E)** Box plot illustrating the odorant-induced reduction in spike frequency of the same seven cells. The odorant concentration was 9% of saturated vapor.

In addition to cells with ON responses we also have encountered neurons with OFF responses. These cells responded with a transient increase in [Ca^2+^]_*i*_ to the offset of an odorant application (Figure 4A). Experiments with three different stimulus durations verified that the OFF cells responded to the offset of an odorant rather than to the onset of the odorant with a long delay.

The third class of responses was observed in cells which decreased their [Ca^2+^]_*i*_ at the onset of odorant application (e.g., cell 2 in Figure 4B). This third class of responses was termed INHIBITED. INHIBITED signals typically had a slow onset but often rapidly returned to the baseline when odorant application was terminated. We hypothesized that INHIBITED neurons are cells which are continuously active and whose activity was suppressed by odorant presentation. Simultaneous two-photon imaging/loose-patch recording confirmed this assumption. All odor-inhibited neurons tested (*n* = 7) were indeed continuously active (Figures 4C,D) and an application of an odorant caused a significant decrease in the firing rate of the neuron or even a complete blockade of cell firing (*p* = 0.001, Mann–Whitney test; the median decrease was 88%; Figure 4E). The reduced spike rate was accompanied by a decrease in [Ca^2+^]_*i*_ (Figure 4C). Odor-inhibited neurons rapidly resumed firing after termination of odorant application. In some neurons (*n* = 4/7) termination of odorant application caused a rebound increase in spiking frequency beyond the prestimulus level (Figure 4C). However, the magnitude of this increase varied between individual cells (not shown) or even between the individual odor-evoked responses of a given neuron (e.g., Figure 4C). There was considerable overlap in the background spike rates of ON and INHIBITED cells suggesting that the type of odorant response (ON or INHIBITED) is determined by other factors.

**Figure 5:**
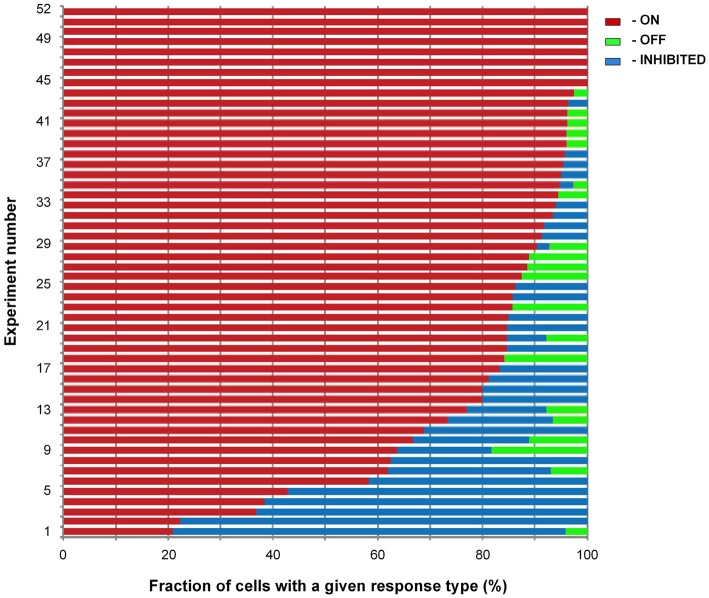
**Distribution of response types.** Summary bar chart illustrating the fraction of cells with a given response type in measurements at 52 locations in 32 preparations (8–51 responding cells per location, median 19 cells). The odorant concentration was 9% of saturated vapor. The fields of view were chosen to contain predominantly cell bodies of juxtaglomerular neurons and came from regions selected for relatively high odorant responsiveness. 1073 out of the 2554 cells present in the 52 locations had a response to the odorant. Eight of the 52 locations had only ON responses; none had only OFF or INHIBITED responses.

We tabulated the occurrence of the three types of responses (ON, OFF, and INHIBITED) in 52 locations in 32 mice (odorant concentration: 9% of saturated vapor). 1073 of the 2554 cells (42%) had responses to the tested odorant. 85% of the responding cells had ON signals, 12% had INHIBITED signals, and 3% OFF signals (Figure 5). Odorant responses from 91 responding glomerular regions had a similar distribution of response types; 87% were ON, 11% were INHIBITED, and 2% had OFF responses.

### Concentration dependence of ON responses

**Figure 6:**
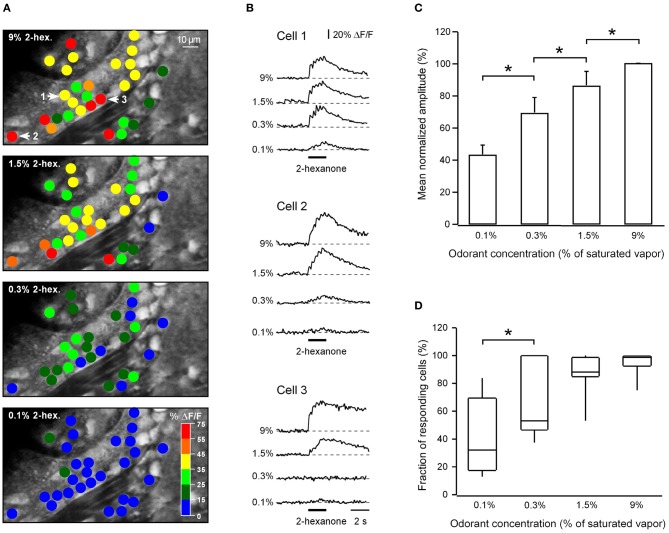
**Concentration dependence of odor-evoked calcium signals. (A)** ON response activity maps for a single plane of focus at four different odorant concentrations. Peak response amplitudes of Ca^2+^ transients of individual cells are displayed using the color code shown in the bottom panel. **(B)** Odor-evoked Ca^2+^ transients recorded from three representative neurons (marked by numbers and arrows in the top panel in **A**). Note that neighboring neurons can have very different odorant sensitivity (e.g., cells 1 and 3). The odorant was 2-hexanone. **(C)** The relationship between the mean normalized amplitude of Ca^2+^ transients and the odorant concentration (*n* = 441 cells in nine experiments). In each experiment the amplitudes of responding cells were averaged for each concentration and normalized to the mean amplitude at 9% of saturated vapor. All concentration-dependent changes in the mean amplitude are significant (^*^, from left to right *p* = 0.02, 0.01, 0.003; Mann–Whitney test). **(D)** Box plot illustrating the fraction of responding neurons at a given odorant concentration (*n* = 9 experiments; from left to right *p* = 0.03^*^, 0.1, 0.06; Mann–Whitney test). To be counted as responding a cell had to show Ca^2+^ transients in response to a given odorant at any concentration tested. The cells which never responded to the odorant were excluded from this analysis. In both **(C,D)** at the lowest concentration tested the numbers will be somewhat distorted by the use of the 15% threshold.

In nine experiments we analyzed how odor-activated cells respond to different odorant concentrations (ranging from 0.1% to 9% of saturated vapor). One such experiment is illustrated in Figures 6A,B. In this experiment only two cells responded to 0.1% of saturated vapor. An increase in odorant concentration was accompanied both with an increase in the amplitudes of odor-evoked Ca^2+^ transients in individual cells and a recruitment of additional responding neurons (Figures 6A–D).

Using loose-seal cell-attached recordings we examined how the spike rate of ON odor-activated neurons changed with an increase in odorant concentration. To quantify odorant-induced changes we measured the number of action potentials per respiration cycle just before as well as during odorant application. In the absence of odorant different ON cells had very different spiking frequencies ranging from 0.1 to 19.6 action potentials per respiration cycle [cells that were silent at rest (*n* = 2) were excluded from this analysis]. Despite these differences, application of an odorant resulted on average in ~60% increase in the spike rate per increase in odorant concentration by 0.7 log of saturated vapor (*n* = 14 cells).

**Figure 7:**
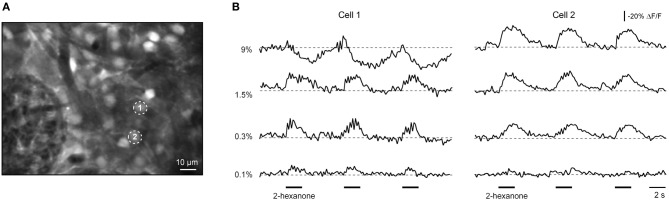
**Response type consistency as a function of odorant concentration. (A,B)** ΔF/F traces recorded from two neurons marked with numbers in **(A)** (as indicated) at different odorant concentrations. Cell 1 changed its response from ON to INHIBITED upon an increase in odorant concentration from 1.5% to 9% of saturated vapor. Cell 2′s response type was the same at all four odorant concentrations. The odorant was 2-hexanone.

Interestingly, some cells exhibiting ON responses at lower odorant concentrations changed their response type to INHIBITED when a higher concentration of the odorant was applied (Figure 7, cell 1). In total, 8 out of 492 ON cells tested at different odorant concentrations changed their response type to NHIBITED. For all of these the switch in the response type occurred at concentrations higher than 1.5% of saturated vapor. Consistent with that result, among neurons tested at 9% of saturated vapor (*n* = 497 cells showing an odorant response) 2.8-times more cells had NHIBITED responses compared to the population (*n* = 391 cells) tested at 1.5% of saturated vapor. These data imply that the type of neuronal response is not determined by specific anatomical connections, but rather originates dynamically from the interplay between excitatory and inhibitory inputs.

### Clustering of odor-responsive neurons near active glomeruli

**Figure 8:**
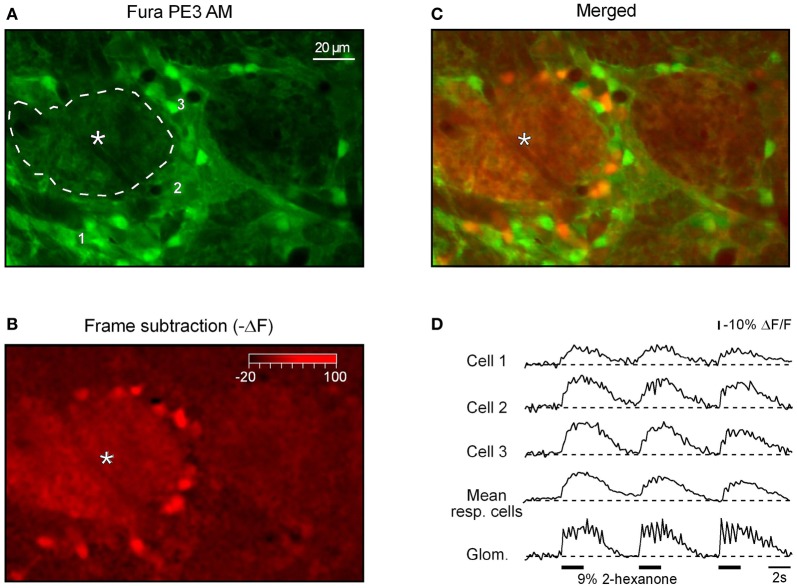
**Spatial distribution of odor-activated cells and glomeruli. (A)** Fura PE3 image of the odor-activated glomerulus (asterisk) and surrounding juxtaglomerular cells and glomeruli. **(B)** shows the -ΔF (frame subtraction) image obtained by subtracting an average of 10 frames recorded at the peak of the odor-evoked response from a similar image recorded before the odorant presentation (2-hexanone, 9% of saturated vapor) and **(C)** shows a merged image. **(D)** Odor-evoked Ca^2+^ transients recorded from a region of interest covering the active glomerulus (delineated by the broken line in **A**) as well as the three cells marked with respective numbers in **(A)**. The trace labeled with mean resp. cells shows an average of ΔF/F traces recorded from all responding neurons (*n* = 16, see Figure A4). The odorant was 2-hexanone. The same data set as the one used to prepare Figures 4A,C in Fink et al. (2012).

**Figure A4:**
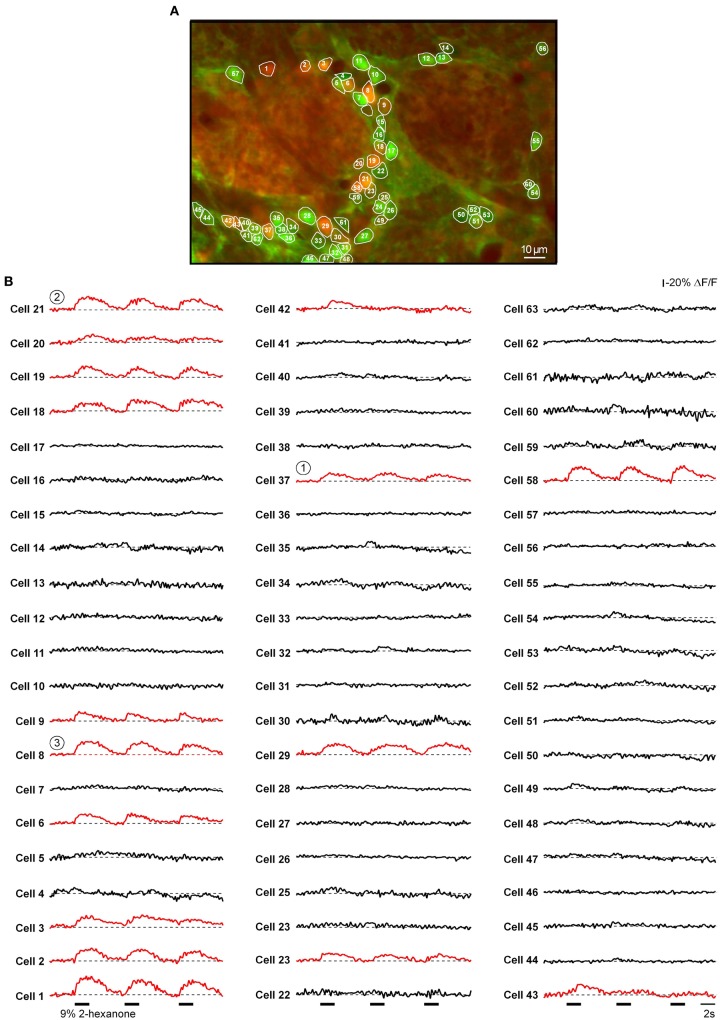
**Odor-evoked calcium signals of all cells located in this plane of focus (the same experiment as the one shown in Figure 8A). (A)** The same image as in Figure 8C. The regions of interest delineate all juxtaglomerular neurons identified in this experiment. **(B)** Odor-evoked Ca^2+^ transients recorded from all cells marked with respective numbers in **(A)**. Circled numbers mark cells illustrated in Figure 8. Red traces correspond to responding cells (identified by automatic signal detection algorithm, see Methods). The average of these is shown in Figure 8D.

Next we asked where the odor-activated cells are located relative to odor-activated glomeruli. Figure 8 shows a fluorescence image of a frame containing both glomeruli and cellular regions. The frame subtraction image (Figure 8B) shows an increase in [Ca^2+^]_*i*_ in the glomerular region indicated by an asterisk. This glomerular region is surrounded by cell body sized regions with increased [Ca^2+^]_*i*_. The merged image (Figure 8C) identifies these regions as ON neurons adjacent to the activated ON glomerulus. The odorant responses of the activated glomerulus and three of the cells (indicated in Figure 8A) are shown in Figure 8D (see Figure A4 for odorant responses of all cells located in this plane of focus). The glomerular signal has clear respiratory modulation which is greatly reduced in the signals from the individual cells. Similar differences in respiratory modulation between glomeruli and cells were seen in 11 out of 12 additional examples that we examined.

**Figure 9:**
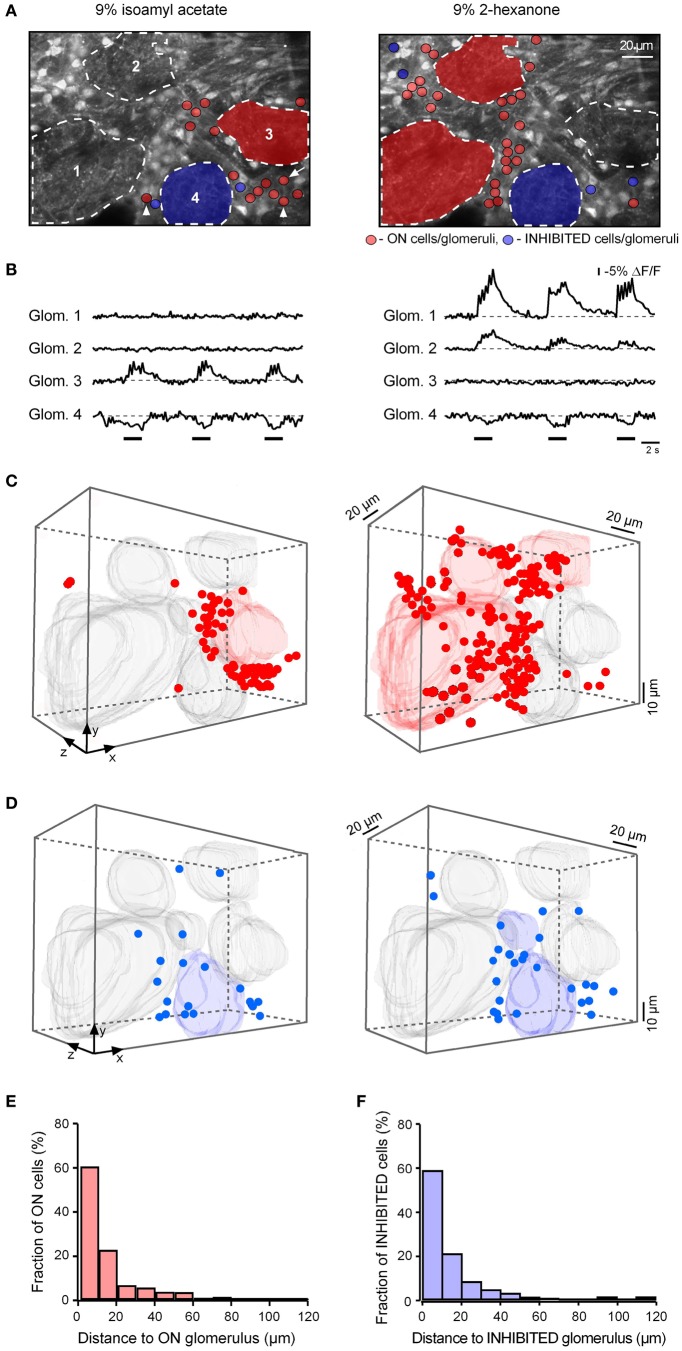
**Clustering of odor-responsive neurons near active glomeruli. (A)** Images of the recorded area. Broken lines delineate odor-responsive glomeruli. Glomeruli 1 and 2 had an ON response to 2-hexanone but no detectable response to isoamyl acetate. In contrast, glomerulus 3 had an ON response to isoamyl acetate but no detectable response to 2-hexanone. Red shadings delineate the glomeruli with ON responses. Glomerulus 4 had an INHIBITED response to both odorants and is colored blue. The arrowheads point toward cells responding to both odorants and an arrow points toward the cell which changed its response from ON to INHIBITED when switching from isoamyl acetate to 2-hexanone. **(B)** Fluorescence recordings (−ΔF/F traces) from regions of interest corresponding to the four glomeruli (marked with corresponding numbers in **A**) during three consecutive applications of isoamyl acetate (left) and 2-hexanone (right). **(C)** The pair of measurements illustrated in **(A,B)** was repeated at eight different depths separated by 10 μm. 3-D volume reconstructions show the location of the ON glomeruli as well as that of the cells activated by isoamyl acetate (left) and 2-hexanone (right). All other glomeruli present in the recorded volume are shown in gray. **(D)** Similar 3-D volume reconstructions as in **(C)** showing INHIBITED cells and glomeruli. **(E)** Histogram summarizing the distance to the three-dimensionally nearest odor-activated glomerulus of all odor-activated ON neurons recorded at seven different planes of focus in this experiment (*n* = 110 cells) as well as in six additional experiments (790 cells in total). **(F)** Histogram summarizing the distance to the nearest odor-inhibited glomerulus of all INHIBITED neurons recorded in five different experiments (89 cells).

To obtain a more complete recording of activity in a given glomerular region, we imaged the cells in several (5–9) planes of focus separated by 8–10 μm through the active glomerulus. Figure 9A illustrates one plane from such an experiment. In this experiment glomeruli 1 and 2 had selective ON responses to 2-hexanone, glomerulus 3 had a selective ON response to isoamyl acetate while glomerulus 4 had an INHIBITED response to both odorants (Figures 9A,B). The selective responses allowed us to determine the location of the responding cells relative to the glomeruli with the same odorant selectivity. Most of the juxtaglomerular cells with ON responses to a specific odorant (red dots) were clustered near glomeruli with the same odorant selectivity (Figures 9A,C). The cell clusters activated by different odorants showed surprisingly little spatial overlap even in the case when different odorants activated neighboring glomeruli (Figures 9A,C, and Movie S3). Out of 207 responding cells only 8 cells (2 of them are marked by arrowheads in Figure 9A) were activated by both odorants in this data set.

**Figure A5:**
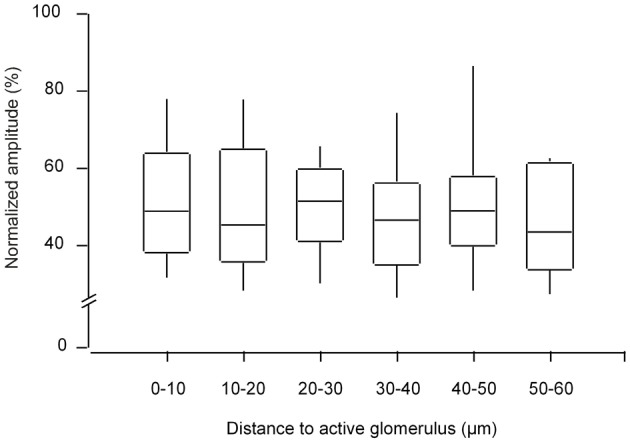
**The amplitude of odorant-evoked signals in the responding cells vs. the distance to the active glomerulus.** Box-Whisker-Plot illustrating the distribution of normalized amplitudes of odor-evoked Ca^2+^ transients plotted with respect to the distance of the cell to the three-dimensionally nearest odor-activated glomerulus (*n* = 790 cells, 7 experiments). In each experiment the amplitudes of odor-evoked Ca^2+^ transients were normalized to the largest recorded value.

**Figure A6:**
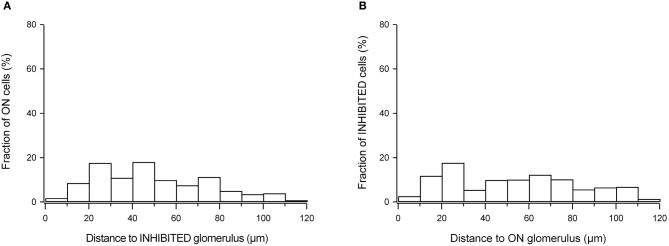
**Distance between odor-responsive cells and glomeruli with different odorant specificity. (A)** Histogram summarizing the distance of all odor-activated ON neurons to the three-dimensionally nearest glomerulus with INHIBITED response (*n* = 5 experiments, 596 cells). **(B)** Histogram summarizing the distance of all INHIBITED neurons to the three-dimensionally nearest glomerulus with ON response (*n* = 5 experiments, 89 cells).

To test whether the response amplitude of the responding cells changes with the distance from the active glomerulus, we analyzed the amplitudes of all recorded cells (*n* = 790 cells in seven experiments), normalizing the values measured in a given experiment to the largest recorded amplitude and plotted these against the distance to the three-dimensionally nearest active glomerulus. This analysis did not reveal any relationship between the signal size and distance (Figure A5). Further, in these seven preparations we measured the three-dimensional distance between ON neurons and the three-dimensionally nearest glomerulus with an ON response. The population data (Figure 9E), clearly shows that ON juxtaglomerular cells are located close to the nearest odor-activated glomerulus with an ON response. In contrast, no such clustering was observed when plotting the distance between ON neurons and the three-dimensionally nearest glomerulus with an INHIBITED response (Figure A6A).

**Figure 10:**
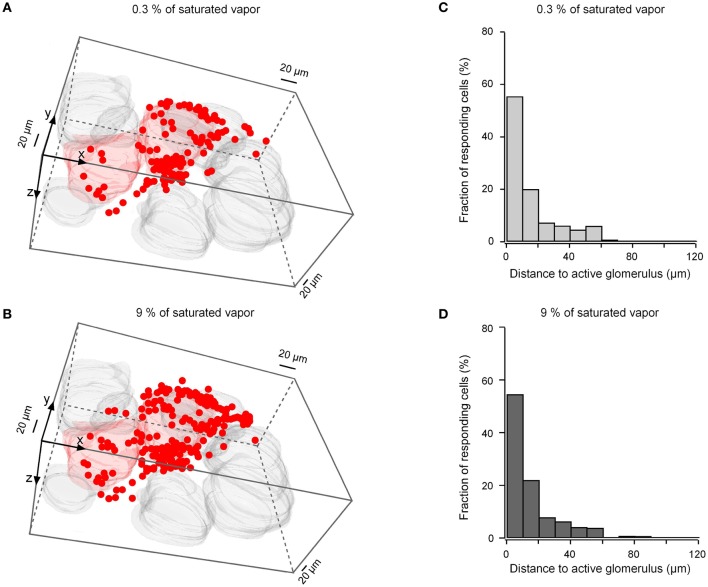
**Concentration dependence of the cluster dimensions. (A,B)** 3-D volume reconstructions of cells and glomeruli activated by 2-hexanone at 0.3% of saturated vapor (**A**, 113 cells, two glomeruli) and at 9% of saturated vapor (**B**, 204 cells, two glomeruli). Because the ON glomerulus in the lower left corner was at the edge of the recorded frame, we might have missed some ON cells surrounding this glomerulus. **(C,D)** Histograms summarizing the position of ON neurons at the two different odorant concentrations with respect to the three-dimensionally nearest ON glomerulus [*n* = 251 cells in **(C)** and *n* = 489 cells in **(D)**, five experiments].

To determine whether the spatial dimension of odor-activated cell clusters changes with odorant concentration we compared odor-evoked activity maps recorded at low (0.3% of saturated vapor) and high (9% of saturated vapor) odorant concentrations. Although the number of responding glomeruli usually increased with an increase in the odorant concentration (Wachowiak and Cohen, 2003; Bozza et al., 2004; Fletcher et al., 2009), we often observed isolated responses of one (e.g., Figure 9C left panel) or a few (Figure 10B) individual glomeruli even at 9% of saturated vapor (Fletcher et al., 2009). In measurements from regions with isolated responses in five preparations, in which both odorant concentrations were tested at several imaging depths, 251 cells responded to the low and 489 cells responded to the high odorant concentration. Despite the approximately 95% increase in the number of responding cells, no increase in the cluster dimensions was observed (Figure 10). Thus, an increase in the odorant concentration causes a recruitment of additional cells to the odor-activated cluster but it does not influence the spatial dimensions of the cluster.

We also analyzed the spatial location of the INHIBITED cells relative to odor-inhibited glomeruli. A three-dimensional reconstruction of the set of data illustrated in Figure 9D shows that for both odorants tested in this experiment most of the INHIBITED cells (blue) were located close to glomeruli with INHIBITED responses. Similar observations were made in five additional experiments (Figure 9F). As for ON neurons, we did not observe clustering of INHIBITED cells around the three-dimensionally nearest glomeruli with ON responses (Figure A6B).

**Figure A7:**
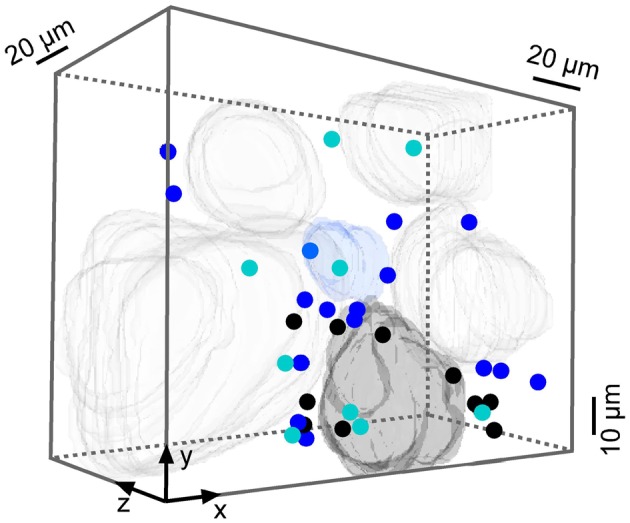
**INHIBITED neurons responding to both odorants.** 3-D volume reconstruction showing cells and glomeruli inhibited by isoamyl acetate (cyan), 2-hexanone (blue), or both (dark gray).

In the experiment shown (Figures 9A,D), approximately half of the INHIBITED cells (53% of cells in the left panel and 38% of cells in the right panel) had inhibited responses to both odorants (Figure A7). The remaining INHIBITED cells were either recruited from non-responding cells or changed their response type from ON to INHIBITED when the odorant was changed (26% of cells in the left panel and 8% of cells in the right panel of Figure 9D changed response type; see also Movies S1, S2).

Taken together, these data show that both ON and INHIBITED juxtaglomerular neurons are not distributed randomly throughout the glomerular layer but rather cluster in the immediate neighborhood of glomeruli with the same odorant response.

## Discussion

This study provides the first comprehensive view into odor-response properties of juxtaglomerular neurons. We show that juxtaglomerular neurons code for many perceptual characteristics of the olfactory stimulus including the identity of the odorant, odorant concentration as well as onset and offset of the odorant application (e.g., its duration). Interestingly, odorant concentration is coded both at the level of individual neurons (as a relative change of the neuron's spike rate) and at the population level (with more cells responding to higher odorant concentrations). Furthermore, our data show that odor-responsive neurons (both activated and inhibited by the odorant) cluster in the close vicinity of their “parent” glomerulus. The size of the odor-responsive cell cluster was unchanged even when the number of odor-responsive cells changed substantially with an increase in the odorant concentration.

### *In vivo* properties of individual juxtaglomerular cells

The majority (~90%) of odor-responsive juxtaglomerular neurons analyzed in this study fired action potentials in the absence of an odorant stimulus. This background activity can either be “sensory-driven” or occur spontaneously. The spontaneous activity of juxtaglomerular neurons can be entrained by spontaneous firing of olfactory sensory neurons (Duchamp-Viret et al., 1999; Tan et al., 2010) and/or can result from intrinsic/synaptic activity within the local glomerular network (Hayar et al., 2004; Pignatelli et al., 2005; Liu and Shipley, 2008; De Saint Jan et al., 2009; Stakic et al., 2011). The “sensory-driven” background activity could result from mechanosensitive properties of receptor neurons involved in monitoring the mouse's respiration (Grosmaitre et al., 2007; Carey et al., 2009). Our results indicate that this background activity is strongly modulated by respiration.

We found three functionally distinct types of odor-evoked responses in juxtaglomerular neurons-ON, OFF, and INHIBITED. INHIBITED responses are seen in ~5% of individual mammalian ORNs (Duchamp-Viret et al., 1999). However, inhibited signals have never been observed in the Ca^2+^ and pH recordings from receptor neuron axon terminals in olfactory bulb glomeruli (Wachowiak and Cohen, 2001; Bozza et al., 2004; Wachowiak et al., 2004; McGann et al., 2005; Vucinic et al., 2006). We conclude, therefore, that odorant suppression of spontaneous ORN activity is unlikely to have a large effect on postsynaptic targets in the bulb. Thus it is likely that only ON responses can be generated as a simple reflection of the input from the nose. OFF and INHIBITED responses are most likely to accrue within the glomerular network.

There was considerable overlap in the background spike rates of ON and INHIBITED cells suggesting that the type of odorant response is not determined solely by the background spiking frequency. Moreover, some individual neurons changed their response type when challenged with two different odorants, or even with the same odorant at a different concentration (e.g., Figure 7). This data suggests that the type of odor-evoked response in a given cell is neither defined by intrinsic properties of the cell nor is it determined by fixed synaptic connections. Rather, it reflects the neuron's response to currently active inhibitory and excitatory inputs.

Individual odor-responsive neurons had very different odorant sensitivity. Some cells have shown clear Ca^2+^ transients in response to the lowest odorant concentration tested (0.1% of saturated vapor), whereas others had undetectable signals even at higher odorant concentrations (Figure 6B). As a population, the juxtaglomerular neurons had a rather wide dynamic range spanning at least two orders of magnitude of odorant concentration. These findings are in line with data obtained when recording electrical activity of mitral/tufted cells (Davison and Katz, 2007; Tan et al., 2010). The majority of the recorded mitral/tufted cells had dynamic range spanning two to three orders of magnitude of odorant concentration (e.g., Figure 3B in Davison and Katz, 2007). In some cases, however, these cells showed extremely wide dynamic range reaching (as a neural population) up to six orders of magnitude (e.g., I7 mitral/tufted cells responding to heptanal; figure 3I in Tan et al., 2010).

Our electrophysiological data suggest that on the level of single cells odorant concentration is coded as a relative increase in the neuron's spike frequency. Coded in this way, odorant concentration can be similarly sensed by juxtaglomerular neurons with very different background spike rates. On the population level there is a net concentration-dependent increase in the number of responding cells. At higher concentrations, however, some ON neurons change their response type and become INHIBITED. This inhibition may be a sign of response suppression at high odorant concentrations designed to avoid saturation of the odorant response amplitudes in output neurons. This would suggest that cells that are INHIBITED at higher odor concentrations are likely to be excitatory neurons.

### Ihibited glomeruli

We have observed clear odor-inhibited Ca^2+^ signals in the glomerular neuropil (e.g., Figure 9B). The inhibited glomerular signals are likely to have a postsynaptic origin because such responses have never been observed in the glomerular Ca^2+^ and pH recordings from receptor neuron axon terminals (Wachowiak and Cohen, 2001; Bozza et al., 2004; Wachowiak et al., 2004; McGann et al., 2005; Vucinic et al., 2006). However, INHIBITED Ca^2+^ signals were found in juxtaglomerular (this study) and mitral/tufted (Lin et al., 2007; Chen et al., 2009) cells. INHIBITED glomerular signals are likely to arise from the glomerular processes of the inhibited juxtaglomerular and mitral/tufted neurons. This finding indicates that net glomerular signals can be dominated by postsynaptic components.

### Clustering of odor-responsive juxtaglomerular neurons

Our mapping data show that the majority of odor-responsive juxtaglomerular cells are clustered within less than 20 μm of the border of an active glomerulus with the same odorant response (“parent” glomerulus). Such an essentially uniglomerular activation pattern is unexpected, and cannot be inferred from the morphological data. Indeed, out of the three major types of juxtaglomerular neurons (periglomerular cells, short-axon cells, and external tufted cells) only periglomerular cells [~40% of all juxtaglomerular neurons (Panzanelli et al., 2007; Parrish-Aungst et al., 2007; Kiyokage et al., 2010)] project most of their dendritic tree to a single glomerulus (Kiyokage et al., 2010). On the other hand, all short-axon cells spread their dendrites over much large distances contacting up to 40 different glomeruli (Kiyokage et al., 2010). In addition, basal dendrites of at least 30% of external tufted cells also extend over several hundreds of micrometers (Antal et al., 2006). In spite of this structural heterogeneity, the odor-evoked activation pattern of juxtaglomerular neurons shows a marked pattern of cell clustering in the immediate vicinity of the “parent” glomerulus.

This clustering pattern is already apparent at lower odorant concentrations (0.3% of saturated vapor). An increase in the odorant concentration to 9% of saturating vapor almost doubled the number or responding cells, but had no detectable effect on the spatial organization of the cluster. This “close proximity rule” identifies a principal glomerulus with a narrow shell of juxtaglomerular neurons as a basic odor coding unit in the glomerular layer. Because odorants usually activate multiple glomeruli throughout the bulb (e.g., Wachowiak and Cohen, 2001; Woo et al., 2007), the information about odorant identity and concentration is likely to be coded by multiple uniglomerular units each including parent glomerulus with the shell of juxtaglomerular cells. In a chemotopic map of the olfactory bulb ON glomeruli coding for different odorants are located in the immediate neighborhood to each other (this paper, Wachowiak and Cohen, 2001; Soucy et al., 2009). Our data show that even at a relatively high odorant concentration (9% of saturated vapor), such units have surprisingly little spatial overlap (Figure 9 and **Movie S3**).

INHIBITED neurons also clustered around INHIBITED glomeruli (Figures 9D,F). Again, 80% of inhibited neurons were within 20 μm of the glomerular border. There is, however, a fundamental difference between ON and INHIBITED cell clusters and their “parent” glomeruli. In the case of ON clusters activity is initiated by ORNs innervating the “parent” glomerulus whereas in the case of INHIBITED clusters the signal originates within the circuitry of the olfactory bulb.

For both ON and INHIBITED cells there were small fractions of neurons (1.5% and 3.3%, respectively) at larger distances (>60 μm) from the principal glomerulus. These distant neurons may belong to the designated “parent” glomerulus or they may belong to glomeruli that are outside of the recorded frame.

### Functional implications

Lateral inhibition has long been postulated as a mechanism for contrast enhancement in the olfactory system (Rall et al., 1966; Wilson and Leon, 1987; Yokoi et al., 1995; Mori et al., 1999). It was originally suggested that lateral inhibition was mediated by reciprocal dendrodendritic synapses between mitral/tufted and inhibitory granule cells. More recently Aungst et al. (2003) suggested the existence of lateral inhibition circuitry in the glomerular cell layer mediated by short axon cells projecting over long distances and affecting remote glomeruli.

One possibility is that these short-axon cells have excitatory effects (Aungst et al., 2003; Kiyokage et al., 2010) activating GABAergic periglomerular cells which, in turn, inhibit corresponding mitral/tufted cells. In view of our data this hypothesis is unlikely because we rarely observed ON cells more than 60 μ away from ON glomeruli (Figure 9E). However, the absence of distant ON cells would not rule out direct inhibitory effects on neighboring glomeruli via GABAergic periglomerular cells. Such local effects could result in INHIBITED cells/glomeruli which we have observed in this study. If so, the INHIBITED cells/glomeruli would have to be located in the immediate neighborhood of strongly activated ON glomeruli. Although our data suggest that this might be the case (Figures 1C, 9A–D), better controlled experiments (for example, those using optogenetics for selective activation of individual glomeruli) are required to test this hypothesis.

The juxtaglomerular neuronal network is unique in that it is capable of modifying the effects of the incoming odor-evoked activity before the first spikes in the output mitral/tufted cells. Our finding that the vast majority of ON cells are clustered within a few tens of microns of ON glomeruli is consistent with a scheme in which olfactory contrast enhancement mostly occurs intraglomerularly. Given that intraglomerular excitatory and inhibitory processes are driven in parallel by the same sensory inputs, but the inhibitory processes are both more sensitive to that input and saturate at lower activity levels (Gire and Schoppa, 2009; Tan et al., 2010), this model generates robust mitral cell spiking in strongly activated ON glomeruli, but inhibits the output from modestly activated units resulting in contrast enhancement (Cleland and Sethupathy, 2006). This mechanism of contrast enhancement relies on intraglomerular circuitry and is better suited for processing fragmented odor maps [i.e., maps in which multiple sensory units (glomeruli) activated by a given odorant are scattered over the surface of the bulb] than a simple center-surround inhibition scheme (Cleland and Sethupathy, 2006). Whether intraglomerular mechanisms also underlie the appearance of INHIBITED cells/glomeruli is less clear. We have observed a small fraction (8.5%, Figure A6A) of ON cells located within 20 μm of INHIBITED glomeruli. It remains to be determined whether these cells belong to the intraglomerular circuitry of the INHIBITED glomerulus (thus possibly driving intraglomerular inhibition) or to a intraglomerular circuitry of the nearby ON glomeruli (see above).

Recently Cleland and Linster (2012) have suggested that intraglomerular circuitry of ON glomeruli is also exceptionally well suited for decorrelation of overlapping sensory representations caused by similar olfactory stimuli. The authors have compared (1) the lateral inhibition and (2) the intraglomerular inhibition scenario (using otherwise identical computational models) and asked which model is better suited for decorrelation of mitral cells firing to enhance differentiation of similar sensory stimuli. In the case of topographical sensory representations, as found in the visual or in the auditory systems, comparable results were provided by both scenarios. For fragmented sensory maps, however, the intraglomerular inhibition scenario was more powerful.

## Author contributions

R. Homma, L. B. Cohen, Y. Kovalchuk, and O. Garaschuk performed the experiments and analyzed the data. R. Homma, L. B. Cohen, Y. Kovalchuk, A. Konnerth, and O. Garaschuk designed the experiments and prepared the manuscript. All authors approved the final version of the manuscript.

### Conflict of interest statement

The authors declare that the research was conducted in the absence of any commercial or financial relationships that could be construed as a potential conflict of interest.

## Supplementary material

The Movies S1–S3 for this article can be found online at http://www.frontiersin.org/Neural_Circuits/10.3389/fncir.2013.00023/abstract

Supplementary Movie S1 **3D-reconstructions of the set of data illustrated in Figures 9A–D**. The movies illustrate the location of the cells activated (red) or inhibited (blue) during the application of 9% isoamyl acetate (Movie S1) or 9% 2-hexanone (Movie S2) as well as the location of glomeruli with the same odorant selectivity. Continuous black lines mark the top x-y and the left y-z surfaces of the imaging frame.

Supplementary Movie S2 **3D-reconstructions of the set of data illustrated in Figures 9A–D**. The movies illustrate the location of the cells activated (red) or inhibited (blue) during the application of 9% isoamyl acetate (Movie S1) or 9% 2-hexanone (Movie S2) as well as the location of glomeruli with the same odorant selectivity. Continuous black lines mark the top x-y and the left y-z surfaces of the imaging frame.

Supplementary Movie S3 **The movie illustrates the location of the cells activated during the application of 9% isoamyl acetate (red) and 9% 2-hexanone (orange) as well as the location of glomeruli with the same odorant selectivity**. The cells activated by both odorants are shown in brown. Continuous black lines mark the top x-y and the left y-z surfaces of the imaging frame. The same data set as in Movies S1 and S2.
